# Dysgeusia increases the risk for death and other side effects during antineoplastic systemic treatment for solid tumors: a cross-sectional study

**DOI:** 10.4317/medoral.26389

**Published:** 2024-04-14

**Authors:** Paulo Goberlânio de Barros Silva, Giulianna Aparecida Vieira Barreto, Anna Clara Aragão Matos Carlos, Marcela Maria Fontes Borges, Cássia Emanuella Nóbrega Malta, Jennifer Vianna Barbosa, André Alves Crispim, Sérgio Ferreira Juaçaba, Lúcio Flávio Gonzaga-Silva

**Affiliations:** 1 Post-Graduate Program in Oncology, Rodolfo Teófilo College/Ceará Cancer Institute; 2 Student Program in Dental Sciences, Unichristus; 3 Graduate Program in Dental Sciences, Unichristus; 4 Post-Graduate Student Program in Oncology, Rodolfo Teófilo College/Ceará Cancer Institute

## Abstract

**Background:**

Chemotherapy (CT) is a systemic treatment using a combination of antineoplastic drugs, orally or intravenously, that inhibit tumor growth and fast-growing normal cells. Due to its nonspecificity, chemotherapy can cause a series of adverse effects, such as altered taste (dysgeusia), associated with malnutrition and, consequently, other adverse effects in the gastrointestinal tract and increased mortality risk. This study aimed to evaluate the influence of dysgeusia on the incidence of other adverse effects and overall survival during antineoplastic chemotherapy.

**Material and Methods:**

An observational, retrospective, cross-sectional study was conducted using data from the Electronic Health Record system of the Cancer Institute of Ceará over two years. Before the CT session, the multi-professional team evaluated the patient for the presence and severity of adverse effects (AE), using scores from the CTCAE v5.0 scale. Dysgeusia scores were collected and associated with clinical pathological data, with other adverse effects (nausea, vomiting, diarrhea, oral mucositis, anorexia, constipation), and with overall survival. Chi-square and Mantel-Cox log-rank tests were used.

**Results:**

Of 5744 patients evaluated, dysgeusia presented a frequency of 50.6%, being directly associated with female gender (*p*=0.001), overweight (*p*=0.022), high tumor stages (*p*=0.009), a combination of adjuvant and neoadjuvant (*p*=0.010) and four-year survival (*p*=0.030). Dysgeusia frequency was directly associated with diarrhea (*p*<0.001), anorexia (*p*<0.001), oral mucositis (*p*<0.001), nausea (*p*<0.001), constipation (*p*<0.001) and vomiting (*p*<0.001), and inversely associated with fatigue (*p*=0.035).

**Conclusions:**

Dysgeusia during CT increases the risk of other adverse effects and negatively impacts prognosis.

** Key words:**Dysgeusia, chemotherapy, radiotherapy.

## Introduction

Most therapeutic protocols in oncology are based on the combination of surgery, radiotherapy, and chemotherapy. Targeted therapies such as biological and immunological, hormonal, and gene therapies are widely expanding, but chemotherapy remains one of the most widely used treatment modalities, using drugs in general in combination or sequence (1).

Chemotherapy for solid tumors is usually associated with combining one or more antineoplastic agents aiming to interfere with various pathways of cell replication. Efficacy increases with the combination of different drugs and with increasing doses administered. However, along with the increased clinical benefit in controlling tumor growth, these combinations increase the incidence of various adverse effects since both tumor cells and rapidly multiplying normal cells are affected by the antineoplastic drugs (2).

Virtually all patients on antineoplastic therapy experience some significant adverse effect, and about 50-75% of patients receiving chemotherapy may experience changes in taste perception consistent with dysgeusia. Dysgeusia consists of a sensation of distorted taste, metallic or unpleasant taste. This condition negatively affects the patient's food intake and nutritional status and is associated with the tissue nonspecificity of most chemotherapeutic agents (3). In patients with head and neck tumors, combining radiotherapy further aggravates this condition due to cytotoxic and antiproliferative effects when radiation affects the tongue and taste buds (4).

In general, the entire gastrointestinal epithelium is rich in rapidly dividing cells, which are the main target of chemotherapy drugs. Thus, patients undergoing antineoplastic chemotherapy have a high prevalence of gastrointestinal tract (GIT) toxicity. Oxidative stress induced by chemotherapeutic agents results in post-translational modification of ion channels altering neuronal excitability significantly and leading to food aversions (taste buds), increased gastrointestinal motility (diarrhea, nausea, and vomiting), and inflammatory immune dysregulation (oral mucositis) (5).

Difficulty in food intake due to dysgeusia interferes with the caloric and protein profile of chemotherapy patients and is directly associated with cachexia (2). Studies have described that chemotherapy patients tend to ingest less protein (6) and increase the intake of foods that cause diarrhea and increased vomiting (7). Malta and colleagues (6) demonstrated that the control of dysgeusia in women treated with chemotherapy reduces not only anorexia but also the incidence of diarrhea, oral mucositis, and vomiting. Thus, the adverse effects of the GIT seem to be directly associated, becoming complicating factors in reducing food intake, predisposing to weight loss, and consequent increase in the incidence of infections impacting the effectiveness of chemotherapy (8,9).

Thus, considering that dysgeusia during chemotherapy impacts seems to be directly related to other adverse effects of systemic antineoplastic treatment, this study aims to evaluate the incidence and association between dysgeusia, and adverse effects of antineoplastic therapy used in treating solid tumors.

## Material and Methods

- Study Design and Ethical Considerations

An observational, retrospective, cross-sectional, and quantitative study will be carried out using data collection from the Electronic Patient Record (PEP) system that will be evolved concerning adverse effects in the oral cavity, dysgeusia, during chemotherapy treatment at the Haroldo Juaçaba Hospital / Cancer Institute of Ceará (HHJ / ICC) over two years (01.01.2018 to 31.12.2020). The present study has the opinion of the Research Ethics Committee of Faculdade Rodolfo Teófilo / ICC with opinion number 4.062.135.

- Inclusion and exclusion criteria

The professionals of the multi-professional team of the CT outpatient clinic of the HHJ/ICC routinely perform the dysgeusia assessment in patients before each CT session by recording the severity scores in the toxicity scales tool, classifying them based on their degree of severity. Therefore, all assessments performed in the period as mentioned earlier retrieved by this tool were included. Patients undergoing treatment for myeloproliferative disorders or treatment of occult or metastatic disease with unknown primary sites were excluded, as well as records lacking the clinical information necessary for risk factor assessment.

- Adverse effects analysis tool

The Toxicity Scales tool is available in the HHJ/ICC Tasy PEP system. Through this tool, professional assistants, nurses, and pharmacists clinically assess the presence and severity of numerous adverse effects before chemotherapy sessions to screen and minimize complications. The toxicity scales encompass the following adverse effects: Mucositis, Vomiting, Diarrhea, Nausea, Constipation, Anorexia, and Dysgeusia. All patients were graded according to the toxicity scores suggested by the Common Terminology Criteria for Adverse Events (CTCAE) v5.0 (2) scale for adverse effects.

Dysgeusia scores were rated on a 3-point scale: grade 0, no change in taste; grade 1, change in taste with no impact on eating habits; and grade 2, a change in taste with impact on eating habits.

Nausea scores were classified as grade 0 when there is no nausea; grade 1 when there is loss of appetite without change in eating habits; grade 2 when oral intake decreases without significant weight loss, dehydration, or malnutrition; grade 3 when there is inadequate fluid or caloric intake, and the use of feeding tubes or hospitalization is indicated.

Diarrhea scores were classified into grade 0, when there is no increase in the number of bowel movements per day; grade 1, when there is an increase of <4 bowel movements per day above baseline; mild increase in ostomy output compared to baseline; grade 2, when there is an increase of 4 to 6 bowel movements per day above baseline, being moderate increase in ostomy output compared to baseline, limiting instrumental ADL; grade 3, when there is an increase of ≥ 7 bowel movements per day above baseline, hospitalization is indicated, a severe increase in ostomy output compared to baseline, limiting self-care ADL, grade 4: when life-threatening, requiring emergency intervention; grade 5: when death.

Oral mucositis was classified as grade 0 when there is no induction of oral mucositis; grade 1 when there are no or mild symptoms, no intervention is required; grade 2 when there is moderate pain or ulcer, but it does not interfere with oral intake, requiring dietary modification; grade 3, when there is severe pain, interfering with oral intake; grade 4, when there is life-threatening, requiring emergency intervention; grade 5 when there is death.

Anorexia scores were classified as grade 0 when there is no loss of appetite or change in eating habits; grade 1, when there is loss of appetite without change in eating habits; grade 2 when oral intake is altered, however, without significant weight loss or malnutrition, requiring supplementation; grade 3, when there is significant weight loss or malnutrition, requiring feeding tubes or TNM; grade 4, when life-threatening, requiring emergency intervention; grade 5, when death occurs.

The constipation scale was defined as grade 0 when there is no presence of constipation; grade 1 when there is the presence of symptoms occasionally or intermittently, with the use of stool softeners, laxatives, diet modification, or enema; grade 2 when there is the presence of persistent symptoms, with regular use of laxatives or enemas; grade 3, when there is constipation with the indication of manual evacuation, limiting ADL self-care; grade 4, when there is a risk of life, requiring emergency intervention; grade 5 when there is death.

Finally, vomiting scores were grouped into grade 0, when there is no vomiting; grade 1, when no intervention is needed; grade 2, when there is outpatient IV hydration, requiring medical intervention; grade 3, when tube feeding, TNM, or hospitalization is needed; grade 4, when it is life-threatening; grade 5 when there is death.

- Collection of sociodemographic and clinical data

After each medical consultation before chemotherapy, the multi-professional team assigned the following toxicity grades for dysgeusia. These were recorded in the toxicity scale tool and exported to a standard Microsoft Excel spreadsheet containing the number and date of care and the severity grade of the adverse effect.

With the number of visits provided by the toxicity scales tool of the Tasy system, a manual search of the PEP of each visit was performed for the clinical-pathological data of interest. Patients who appeared more than once were sorted by service date to identify the number of the CT cycle being evaluated. During the manual collection of information based on the number of care, the patient's medical record will be collected, as well as age, sex, weight on the day of care, height, intention of chemotherapy (neoadjuvant, adjuvant or palliative), clinical stage, TNM, chemotherapy protocol and location of the primary tumor, in addition to the date of initiation of chemotherapy and date of last visit or death for calculation of overall survival.

In the case of patients with head and neck tumors, information will be collected about previous/concomitant head and neck radiotherapy. All data were tabulated in a Microsoft Excel spreadsheet.

- Statistical analysis

The data were exported to the Statistical Package for the Social Sciences (SPSS) software version 20.0 for Windows, in which the analyzes were performed adopting a 95% confidence level. The prevalence of dysgeusia scores was expressed as absolute and percentage frequency and compared with risk factors using Fisher's exact or Pearson's chi-square tests. Additionally, Kaplan-Meier curves were created to calculate overall survival, which will be compared with adverse effects using the Mantel-Cox log-rank tests. After that, variables with *p*<0.200 were submitted to a multinomial logistic regression model (multivariate analysis) and COX regression.

## Results

A total of 5744 patients were evaluated in this study. Of these, 2,907 (50.6%) patients experienced dysgeusia throughout their chemotherapy protocols for the treatment of solid tumors, of which 1,811 (31.5%) had grade I dysgeusia and 1,096 (19.1%) had grade II dysgeusia (Table 1).

The majority of patients evaluated were female (*n*=3999, 69.6%), which was directly associated with grade I and II dysgeusia (*p*<0.00)1, and aged between 41-60 years (*n*=2603, 45.3%) or 61-80 (*n*=2323, 40.4%), with dysgeusia being significantly more frequent in patients aged between 41-60 years (*p*<0.001). The majority of patients were normotensive during chemotherapy (*n*=2478, 44.6%), and this parameter was not significantly associated with dysgeusia during CT (*p*=0.053) (Table 1).

Most of the primary tumors under treatment were breast tumors (*n*=1926, 33.5%), and patients with colorectal, lung, head and neck, and uterus tumors had a higher frequency of dysgeusia. Of the head and neck tumors, 469 (92.1%) patients had radiotherapy; this parameter was not associated with dysgeusia during chemotherapy (*p*=0.729), and the most frequent stage of tumors was stage IV (*n*=1397, 46.0%).

**Table 1:**
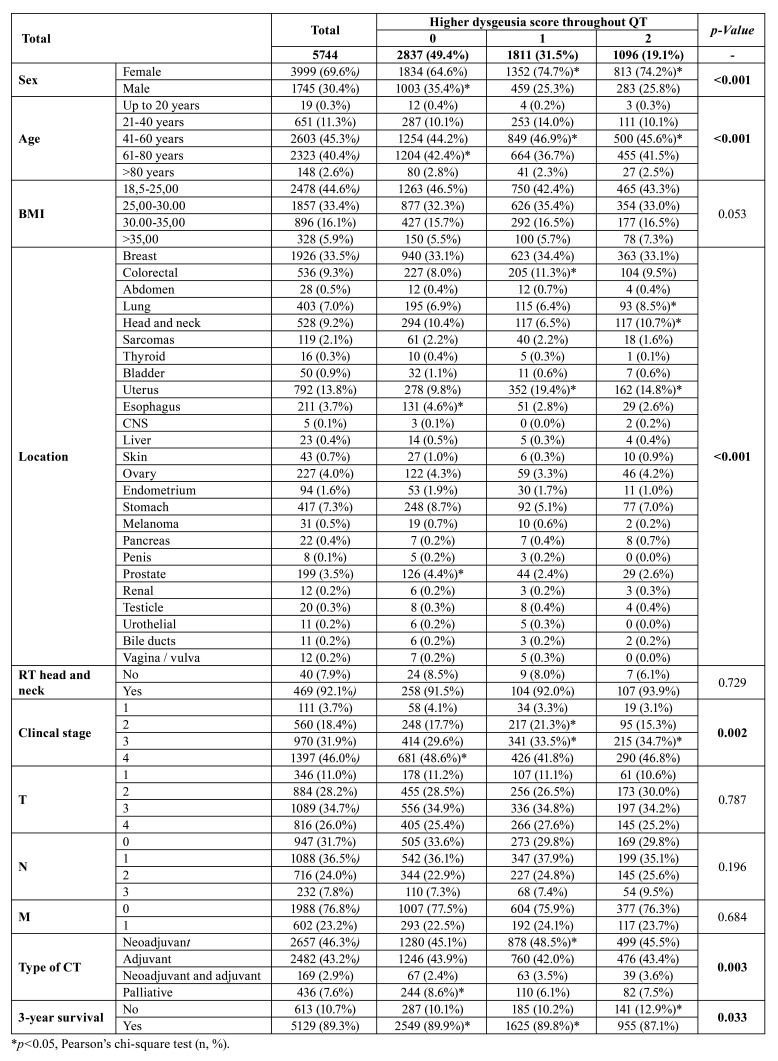
Clinical profile, incidence, and risk factors for dysgeusia during chemotherapy in patients undergoing systemic antineoplastic treatment for solid tumors.

Stages II and III were significantly associated with dysgeusia during chemotherapy (*p*=0.002). The most common T, N, and M stages were T3 (*n*=1089, 34.7%), N1 (*n*=1088, 36.5%), M0 (*n*=1988, 76.8%), and these were not associated with dysgeusia (*p*>0.05) (Table 1).

The most frequent chemotherapies were neoadjuvant chemotherapy (*n*=2657, 46.3%) followed by adjuvant chemotherapy (*n*=2482, 43.2%), with neoadjuvant chemotherapy associated with grade I dysgeusia (*p*=0.003). Of the 5744 patients, 613 (10.7%) died within the first three years of treatment, and this variable was directly associated with grade II dysgeusia (*p*=0.033) (Table 1).

**Figure 1:**
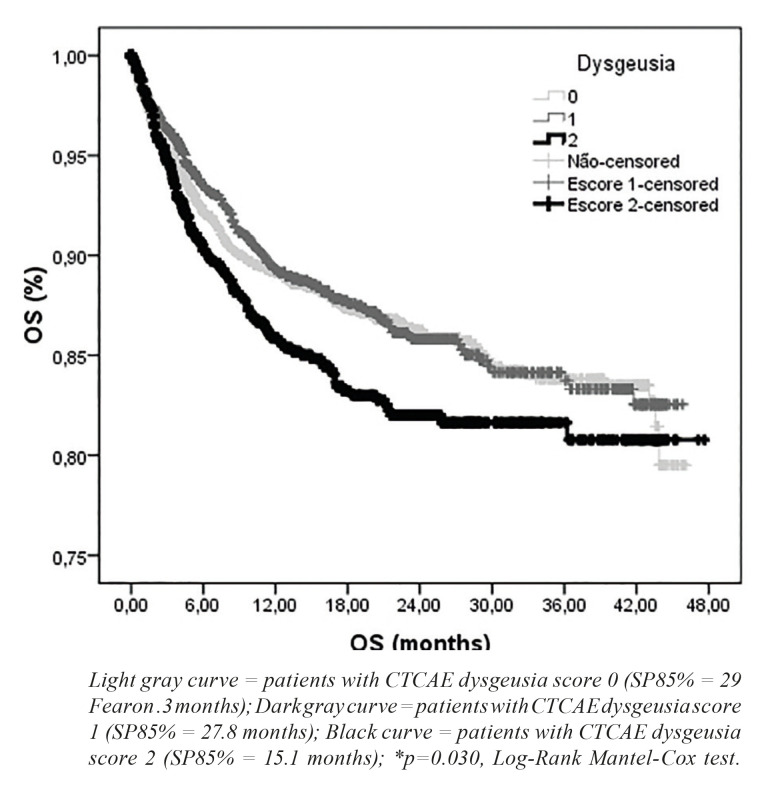
Kaplan-Meier curve of overall survival of patients undergoing dysgeusia assessment during systemic antineoplastic treatment of solid tumors.

The 85% overall survival of the group of patients with dysgeusia score II (SP85% = 15.1 months) was significantly lower than that of patients with maximum dysgeusia score grade 1 (SP85% = 27.8 months) and grade 0 (SP85% = 29.3 months) (*p*=0.030). The presence of dysgeusia increased the risk of death by 8.07% (95% CI = 3.36 - 12.99%) within three years of starting chemotherapy (OR = 1.08, 95% CI = 1.03-1.13) (Fig. 1).

The 5744 patients included underwent 32,925 cycles of chemotherapy, being evaluated in all of these and totaling a mean of 5.5±4.3 evaluations. During these 32,925 chemotherapy cycles, dysgeusia was the most severe, followed by nausea and anorexia (*p*<0.001). Diarrhea, oral mucositis, constipation, vomiting, and fatigue had the lowest severity (*p*<0.001) and lowest frequency (*p*<0.001) (Table 2).

**Table 2:**
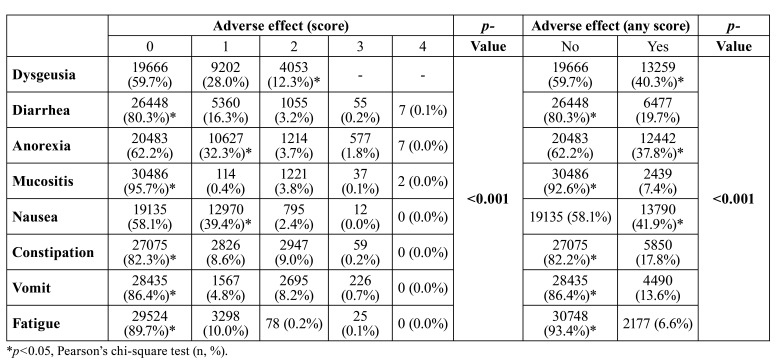
Incidence of dysgeusia and other adverse effects during chemotherapy in patients undergoing systemic antineoplastic treatment for solid tumors.

When dysgeusia was associated with other variables, it was observed that the presence of dysgeusia score I or II was directly associated with chemotherapy cycles (*p*<0.001), female gender (*p*<0.001), high BMI (*p*<0.001), chemotherapy for cervical tumors (*p*<0.001), head and neck radiotherapy (*p*=0.009), nodal metastasis (*p*<0.001) and adjuvant chemotherapy (*p*=0.007) (Table 3).

**Table 3:**
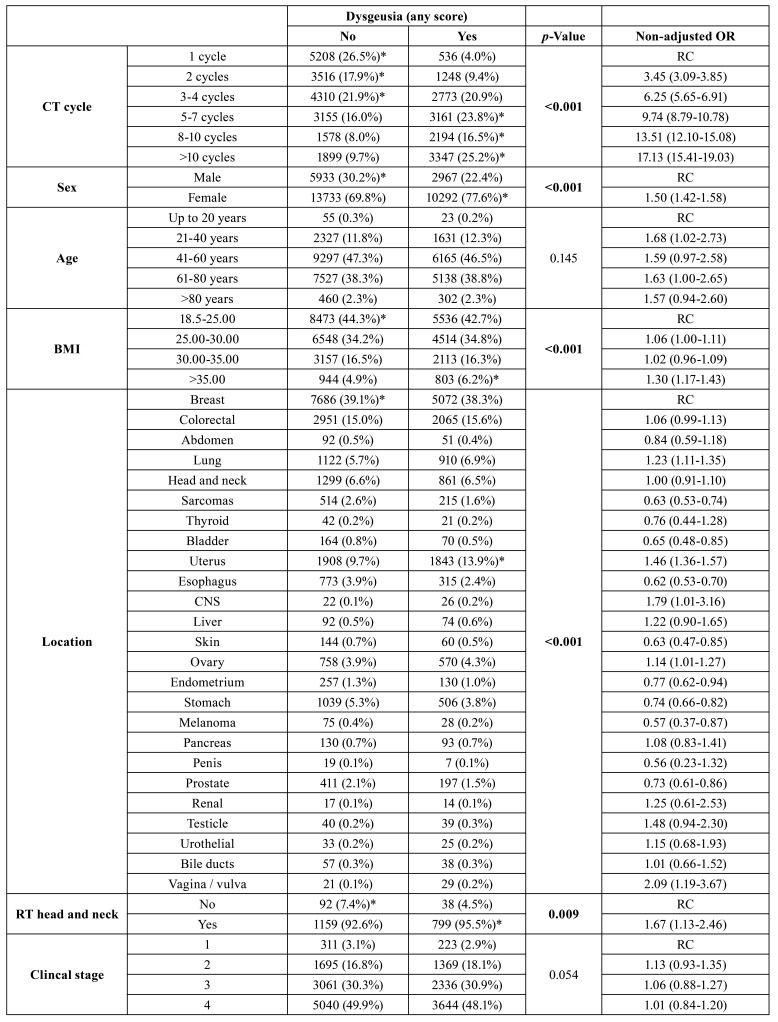
Influence of dysgeusia during chemotherapy on clinical parameters and other adverse effects in patients undergoing systemic antineoplastic treatment for solid tumors.

**Table 3 cont.**
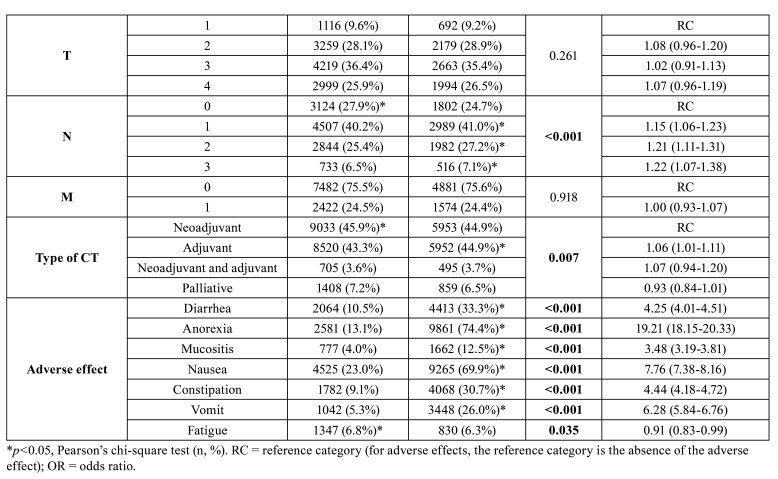
Influence of dysgeusia during chemotherapy on clinical parameters and other adverse effects in patients undergoing systemic antineoplastic treatment for solid tumors.

The frequency of dysgeusia significantly increased the frequency of diarrhea (*p*<0.001), anorexia (*p*<0.001), oral mucositis (*p*<0.001), nausea (*p*<0.001), constipation (*p*<0.001), vomiting (*p*<0.001) and was inversely associated with fatigue (*p*=0.035) (Table 3).

In multivariate analysis, dysgeusia was directly associated with female gender (*p*=0.001), overweight (*p*=0.022), clinical stages higher than 1 (*p*=0.009), T stage higher than T1 (*p*=0.006), adjuvant or neoadjuvant chemotherapy (*p*=0.010), anorexia (*p*=0.001), oral mucositis (*p*=0.032), constipation (*p*=0.019) and death within three years of treatment initiation (*p*=0.030) (Table 4).

**Table 4:**
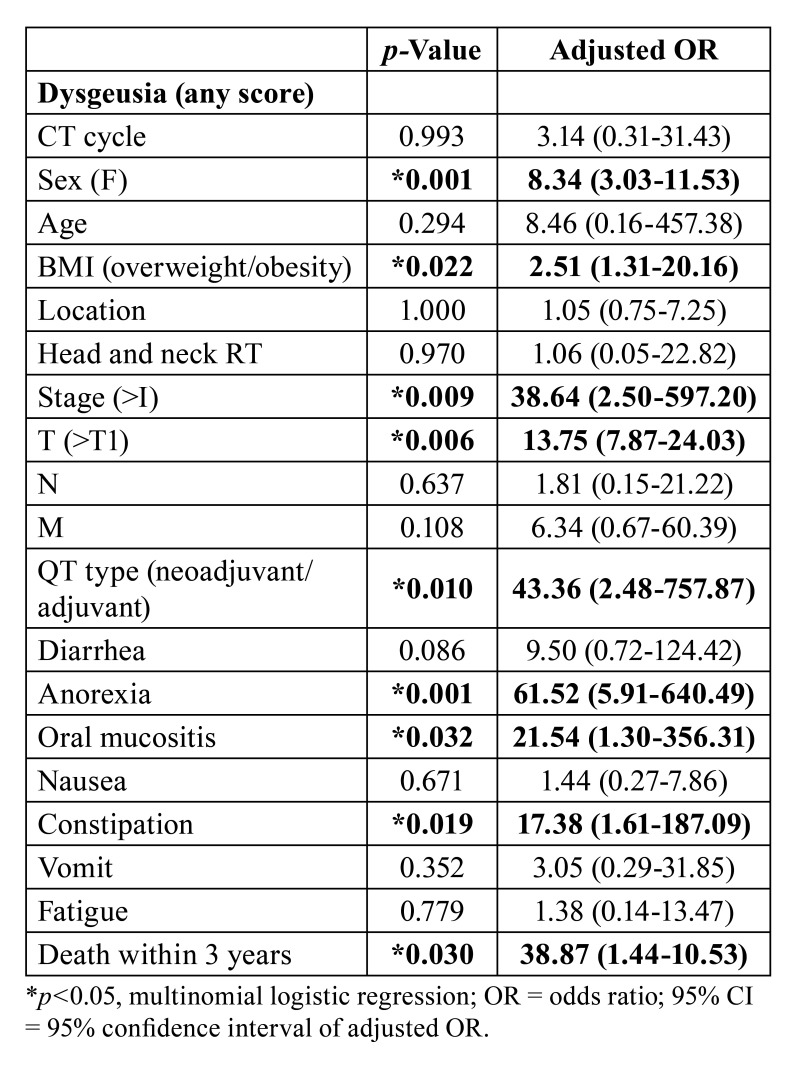
Multivariate analysis of factors associated with dysgeusia during chemotherapy in patients undergoing systemic antineoplastic treatment for solid tumors.

## Discussion

This study showed that approximately 50.6% of chemotherapy patients developed some taste distortion, with 31.5 presenting grade 1 dysgeusia and 19.1% grade 2. These values are well above those described by Malta and colleagues (2) who retrospectively observed dysgeusia in only one-fifth of their sample, below those described by Zbernigg and colleagues (10), in which dysgeusia was reported in about 69.9% of patients undergoing chemotherapy, but within the expected by Denda and colleagues (2020) (11) who demonstrated that about 56% of patients had dysgeusia during chemotherapy. Thus, the frequency of dysgeusia during antineoplastic therapy depends significantly on the study design and the population evaluated.

Female patients represented a risk group for grade 1 and 2 dysgeusia. Malta and colleagues (2) also described this group as a risk group for loss of taste, and it has been suggested a decreased sensitivity of smell and its relationship with the presence of estrogen receptors, justifying before neuroendocrine defects in women or neurotransmitters with hypothalamic origin (12).

Breast cancer was the most prevalent in our study; however, given the large sample, it was not significant when associated with dysgeusia. Although studies have shown that 76.1% of breast cancer patients undergoing chemotherapy had moderate or severe taste alterations (6,12), it should be considered that the advancement of protocols and fractionation of doses of chemotherapy drugs based on the treatment of this tumor, such as doxorubicin, cyclophosphamide, paclitaxel, and docetaxel, are associated with the control of many adverse effects (13).

The tumor at the highest risk for dysgeusia in our study was cervical cancer. Therapeutic protocols for uterine cancer mainly involve drugs from the platinum family, such as cisplatin and carboplatin, which are already demonstrated in the literature with their significant association with changes in taste (14). Other studies have also shown that patients with cervical cancer had a high prevalence of taste alterations, with this adverse effect affecting more than a quarter of their sample (12).

Head and neck tumors have also shown a significant association with dysgeusia since most head and neck tumors receive radiotherapy at some point in their treatment (15). Small doses of radiotherapy can lead to dysgeusia, and alteration in taste perception has been demonstrated in patients who underwent radiotherapy with a dose of less than 30Gy (4). In addition to the cytotoxic effect of radiotherapy on taste buds and oral tissues, it is suggested that hyposalivation also participates in the mechanisms of taste receptor dysfunction (16). Moreover, platinum is the first line of systemic treatment of head and neck cancers, and cisplatin and carboplatin are strongly associated with unpleasant taste sensations (17).

Dysgeusia was significantly more frequent in patients aged 41-60 years. With increasing age, there is a decrease in chemosensory capacity, in addition to impairments in cognitive function and changes in brain activity in regions involved with the cognitive function of patients, which makes older patients a risk group for loss of taste perception compared to younger patients (18).

In our study, the therapeutic intentions of chemotherapy (neoadjuvant and adjuvant) and higher-stage tumors were risk factors for dysgeusia. Neoadjuvant and/or adjuvant protocols generally involve higher chemotherapy doses than palliative therapies, where quality of life and comfort are the main objectives (19). Thus, higher doses of chemotherapy are expected in these treatment modalities and, consequently, higher frequency of dysgeusia and adverse effects (12,20).

Dysgeusia was also associated with several adverse effects on the gastrointestinal tract, including diarrhea, anorexia, mucositis, nausea, constipation, and vomiting. The GIT is vital for digestion, nutrient absorption, and waste excretion (21). Throughout its distribution, there is the presence of cells and neurons that will act by regulating intestinal motility, as well as by exciting or inhibiting smooth muscle, in addition to having sensory pathways commonly associated with transmitting signals to the central nervous system (22). Despite the great advances in antineoplastic treatment, evidenced by chemotherapy, there is still a significant prevalence of adverse effects, especially in the GIT, such as nausea, vomiting, constipation, and diarrhea, which can significantly interfere with the continuity of treatment (21). A recent study showed that a decreased sensation of taste, lower appetite, less hunger, dry mouth, and nausea were associated with a lower caloric intake, which limits the pleasure of eating, making eating more difficult, thus causing a nutritional deficit in cancer patients (3).

In particular, the association with nausea and vomiting causes growing concern since dysgeusia and nausea and vomiting are related to food aversion, given the altered sensation of flavor (7,22,23). Decreased taste sensation generates less appetite, hunger, dry mouth, and nausea, and these effects are associated with lower food intake and nutritional deficit in cancer patients (24). Malnutrition is common in patients undergoing antineoplastic treatment and may be directly associated with cancer or chemotherapy (25).

Given the food aversion promoted by the alteration in taste, and a decrease in hunger, malnutrition in the patient may increase the risk of death. We observed that dysgeusia is independently associated with anorexia and lower overall survival, a serious association also described by Prieto-Callejero and colleagues (26), who described that weight loss is a strong predictor of prognosis in cancer patients. Dysgeusia, combined with other adverse effects, mainly gastrointestinal ones, increases malnutrition and weight loss in cancer patients, potentially affecting the response and tolerance to chemotherapy treatment, decreasing quality of life, and associating with worse survival of patients (27).

Other adverse effects, such as oral mucositis, constipation, and diarrhea, are also associated with dysgeusia. The painful lesions of oral mucositis, in addition to interfering with food intake, are often secondarily colonized by bacteria and fungi (28) leading to dysbiosis in addition to interfering with the taste of food (29) modifies the microbiota of the rest of the gastrointestinal tract leading to changes in motility (constipation and diarrhea) (30).

Although this study has the limitation of being retrospective, which makes associations difficult, and numerous professionals perform data collection, it should be emphasized that the entire team is trained to use the CTCAE scale (2) during their care, leading to a significant volume of data, partially circumventing this limitation.

This study showed, therefore, that dysgeusia during antineoplastic chemotherapy, in addition to having a high incidence and well-described risk factors, is associated with increased severe adverse effects in the gastrointestinal tract and the risk of anorexia and death, making it essential to develop studies that develop ways to minimize its incidence.

## References

[1] Chaveli-López B (2014). Oral toxicity produced by chemotherapy: A systematic review. J Clin Exp Dent.

[2] Malta CEN, de Lima Martins JO, Carlos ACAM, Freitas MO, Magalhães IA, de Vasconcelos HCA (2022). Risk factors for dysgeusia during chemotherapy for solid tumors: a retrospective cross-sectional study. Support Care Cancer.

[3] Ponticelli E, Clari M, Frigerio S, De Clemente A, Bergese I, Scavino E (2017). Dysgeusia and health-related quality of life of cancer patients receiving chemotherapy: A cross-sectional study. Eur J Cancer Care (Engl).

[4] Irune E, Dwivedi RC, Nutting CM, Harrington KJ (2014). Treatment-related dysgeusia in head and neck cancer patients. Cancer Treat Rev.

[5] Akbarali HI, Muchhala KH, Jessup DK, Cheatham S (2022). Chemotherapy induced gastrointestinal toxicities. Adv Cancer Res.

[6] Malta CEN, Carlos ACAM, de Alencar MCM, Alves E, Silva EF, Alves APNN (2022). Photobiomodulation therapy prevents dysgeusia chemotherapy induced in breast cancer women treated with doxorubicin plus cyclophosphamide: a triple-blinded, randomized, placebo-controlled clinical trial. Support Care Cancer.

[7] Bozzetti F, Mariani L (2009). Defining and classifying cancer cachexia: A proposal by the SCRINIO Working Group. JPEN J Parenter Enter Nutr.

[8] Fejzo MS, Trovik J, Grooten IJ, Sridharan K, Roseboom TJ, Vikanes Å (2019). Nausea and vomiting of pregnancy and hyperemesis gravidarum. Nat Rev Dis Prim.

[9] Dahlgren D, Rosenqvist E, Hellström PM, Nygren P, Kullenberg F, Peters K (2022). Evaluation and validation of chemotherapy-specific diarrhea and histopathology in rats. Basic Clin Pharmacol Toxicol.

[10] Zabernigg A, Gamper EM, Giesinger JM, Rumpold G, Kemmler G, Gattringer K (2010). Taste alterations in cancer patients receiving chemotherapy: a neglected side effect?. Oncologist.

[11] Denda Y, Niikura N, Satoh-Kuriwada S, Yokoyama K, Terao M, Morioka T (2020). Taste alterations in patients with breast cancer following chemotherapy: a cohort study. Breast Cancer.

[12] Gamper EM, Giesinger JM, Oberguggenberger A, Kemmler G, Wintner LM, Gattringer K (2012). Taste alterations in breast and gynaecological cancer patients receiving chemotherapy: prevalence, course of severity, and quality of life correlates. Acta Oncol.

[13] Gadisa DA, Wang SH, Yimer G (2021). The Impact of AC and AC-T chemotherapy's toxicities on quality of life among women with breast cancer in Ethiopia: A prospective patient-reported outcomes study. Breast Cancer (Dove Med Press).

[14] Amézaga J, Alfaro B, Ríos Y, Larraioz A, Ugartemendia G, Urruticoechea A (2018). Assessing taste and smell alterations in cancer patients undergoing chemotherapy according to treatment. Support Care Cancer.

[15] Mossman KL (1986). Gustatory tissue injury in man: radiation dose response relationships and mechanisms of taste loss. Br J Cancer Suppl.

[16] Epstein JB, Barasch A (2010). Taste disorders in cancer patients: pathogenesis, and approach to assessment and management. Oral Oncol.

[17] Bressan V, Stevanin S, Bianchi M, Aleo G, Bagnasco A, Sasso L (2016). The effects of swallowing disorders, dysgeusia, oral mucositis and xerostomia on nutritional status, oral intake and weight lossin head and neck cancer patients: a systematic review. Cancer Treat Rev.

[18] Pugnaloni S, Vignini A, Borroni F, Sabbatinelli J, Alia S, Fabri M (2020). Modifications of taste sensitivity in cancer patients: a method for the evaluations of dysgeusia. Support Care Cancer.

[19] Rha SY, Lee J (2017). Symptom clusters during palliative chemotherapy and their influence on functioning and quality of life. Support Care Cancer.

[20] Ogata R, Yamamoto Y, Jo A, Fukuma Y, Mikami T, Kawano S (2022). Neoadjuvant Epirubicin-Cyclophosphamide Therapy followed by Nab-Paclitaxel in Patients with Operable Breast Cancer-A Single-Center Phase Ⅱ Trial. Gan To Kagaku Ryoho.

[21] Escalante J, McQuade RM, Stojanovska V, Nurgali K (2017). Impact of chemotherapy on gastrointestinal functions and the enteric nervous system. Maturitas.

[22] Costa M, Brookes S (2008). Architecture of enteric neural circuits involved in intestinal motility. Eur Rev Med Pharmacol Sci.

[23] Hinkelmann JV, Possa LO, de Oliveira CA, Faria BS, Hermsdorff HHM, Rosa COB (2021). Food preferences and aversions of patients undergoing chemotherapy, radiotherapy and/or hematopoietic stem cell transplantation. Clin Nutr ESPEN.

[24] de Vries YC, van den Berg MMGA, de Vries JHM, Boesveldt S, de Kruif JTCM, Buist N (2017). Differences in dietary intake during chemotherapy in breast cancer patients compared to women without cancer. Support Care Cancer.

[25] Agate L, Minaldi E, Basolo A, Angeli V, Jaccheri R, Santini F (2022;18). Nutrition in Advanced Thyroid Cancer Patients. Nutrients.

[26] Prieto-Callejero B, Rivera F, Fagundo-Rivera J, Romero A, Romero-Martín M, Gómez-Salgado J (2020). Relationship between chemotherapy-induced adverse reactions and health-related quality of life in patients with breast cancer. Medicine (Baltimore).

[27] Sánchez-Lara K, Sosa-Sánchez R, Green-Renner D, Rodríguez C, Laviano A, Motola-Kuba D (2010;24). Influence of taste disorders on dietary behaviors in cancer patients under chemotherapy. Nutr J.

[28] Martins JO, Borges MM, Malta CE, Carlos AC, Crispim AA, Moura JF (2022). Risk factors for oral mucositis during chemotherapy treatment for solid tumors: a retrospective STROBE-guided study. Med Oral Patol Oral Cir Bucal.

[29] Chitapanarux I, Wongsrita S, Sripan P, Kongsupapsiri P, Phakoetsuk P, Chachvarat S (2021). An underestimated pitfall of oral candidiasis in head and neck cancer patients undergoing radiotherapy: an observation study. BMC Oral Health.

[30] Nobre LMS, Fernandes C, Florêncio KGD, Alencar NMN, Wong DVT, Lima-Júnior RCP (2023). Could paraprobiotics be a safer alternative to probiotics for managing cancer chemotherapy-induced gastrointestinal toxicities?. Braz J Med Biol Res.

